# Distal sacral nerve roots severed by a fragility fracture of the sacrum: a case report

**DOI:** 10.1186/s13256-022-03551-z

**Published:** 2022-08-23

**Authors:** Shun Igarashi, Takashi Kobayashi, Hiroaki Kijima, Naohisa Miyakoshi

**Affiliations:** 1 Department of Orthopedic Surgery, Akita University Graduate School of Medicine, 1-1-1 Hondo, Akita, Akita 010-8543 Japan; 2 Department of Orthopedic Surgery, Akita Kousei Medical Center, 1-1-1 Nishibukuro Iijima, Akita, Akita 011-0948 Japan

**Keywords:** Sacral fracture, Fragility fracture, Sacral nerve root, Gait disorder, Fecal incontinence, Sacral laminectomy, Low back pain

## Abstract

**Background:**

Owing to the aging population, fragility fractures of the pelvis are occurring more frequently. Fixation of the fracture and stabilization of the pelvic ring usually provide good clinical results. A case of distal sacral nerve roots severed by a fragility fracture of the sacrum is presented.

**Case presentation:**

A 62-year-old Japanese woman with schizophrenia with low back pain, gait disorder, dysuria, and fecal incontinence presented to an emergency department, and plain X-rays showed no findings. She also complained of dysuria, and neurogenic bladder and cystitis were diagnosed. One month later, she was admitted to a psychiatric hospital for exacerbation of schizophrenia. In hospital, she had a urethral catheter inserted and spent 3 months in bed. She was referred to our orthopedic department because a gait disorder was discovered after her mental condition improved and she was permitted to walk. On examination, she could not walk and had decreased sensation from the buttocks to both posterior thighs and around the anus and perineum. Manual muscle testing of her lower limbs showed mild weakness of about 4 in bilateral flexor hallucis longus and gastrocnemius, and bilateral Achilles tendon reflexes were lost. Her anal sphincter did not contract, and urinary retention continued after urethral catheter removal. Imaging examinations showed an H-shaped sacral fracture consisting of a transverse fracture with displacement of the third sacral vertebra and vertical fractures of the bilateral sacral wings, with severe stenosis of the spinal canal at the site of the transverse fracture. The patient was diagnosed as having bladder and rectal dysfunction due to a displaced, unstable sacral fracture. First to third sacral laminectomy and alar–iliac fixation using percutaneous pedicle screws and sacral alar–iliac screws were then performed. The bilateral distal sacral nerve roots (S3, S4, S5) were completely severed at the second to third sacral levels, but bilateral second sacral nerve roots were not compressed from the bifurcation to the sacral foramen. Postoperatively, bladder and rectal dysfunction remained, but the low back pain was alleviated. Two weeks postoperatively, she could walk with a walker and was discharged. Three months after the operation, bone fusion of the fracture was observed.

**Conclusions:**

In cases of bladder–rectal dysfunction with low back pain, the possibility of sacral fracture should be considered, and computed tomography, magnetic resonance imaging, and X-ray examinations should be performed. Even sacral fractures without displacement require attention because they can cause serious injury in the event of a nerve root being severed if not diagnosed early and given appropriate treatment.

## Background

Owing to the aging population, fragility fractures of the pelvis (FFPs) are increasing [1]. The AO (Arbeitsgemeinschaft für Osteosynthesefragen) classification [2], which classifies high-energy trauma, was previously used for FFPs. In addition, the Denis classification [3] is often used to classify sacral fractures in particular. However, a new classification for FFPs was proposed by Rommens *et al*. in 2013 [4]. In recent years, there have been many reports of FFP treatments according to this classification. Moreover, the treatment for FFPs usually gives good clinical results if fixation of the fracture and stabilization of the pelvic ring are obtained. However, a case of distal sacral nerve roots severed by a fragility fracture of the sacrum is presented. Because the surgical treatment succeeded in bringing this patient back into society, this patient’s course of treatment is presented.

## Case presentation

**Fig. 1:**
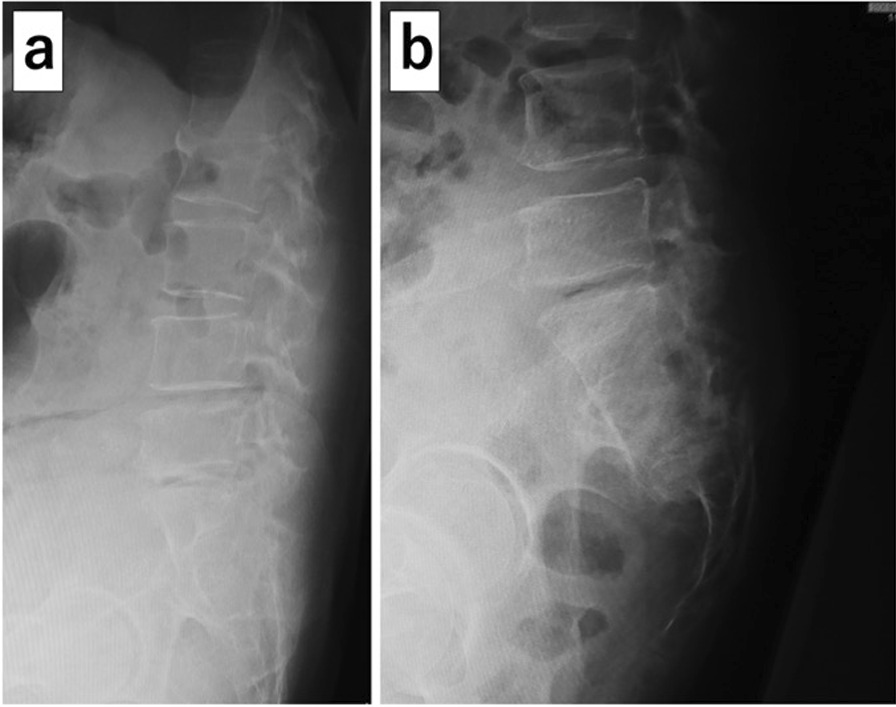
X-ray lateral image. **a** Lateral image of the lumbar spine on admission to the emergency department. Sacral fracture is not evident. **b** Lateral image of the sacrum on admission to the orthopedic department. A transverse fracture of the third sacral vertebra with displacement is shown

A 62-year-old Japanese woman with schizophrenia complained of low back pain, gait disorder, dysuria, and fecal incontinence. She had a history of schizophrenia and chronic obstructive pulmonary disease. She had no previous history of fractures. She first visited an emergency department because of her low back pain that had occurred 4 months earlier and suddenly worsened. However, her low back pain was treated conservatively, because a duty doctor found no significant findings on plain X-ray of her lumbar spine (Fig. 1a). At the same time, she also complained of dysuria, and a urologist diagnosed neurogenic bladder and cystitis. One month later, she was admitted to a psychiatric hospital for exacerbation of schizophrenia. At the hospital, she had a urethral catheter inserted because of difficulty urinating on her own. She spent 3 months in bed until her schizophrenic symptoms improved.

She was referred to our orthopedic department because a gait disorder was discovered after her mental condition improved and she was permitted to walk. At the first visit to our department, she could not walk and had loss of sensation from the buttocks to both posterior thighs and around the anus and perineum. Manual muscle testing of her lower limbs showed mild weakness of about 4 in bilateral flexor hallucis longus and gastrocnemius, and bilateral Achilles tendon reflexes were lost. Her anal sphincter was unable to contract, and urinary retention continued after removal of the urethral catheter.

**Fig. 2:**
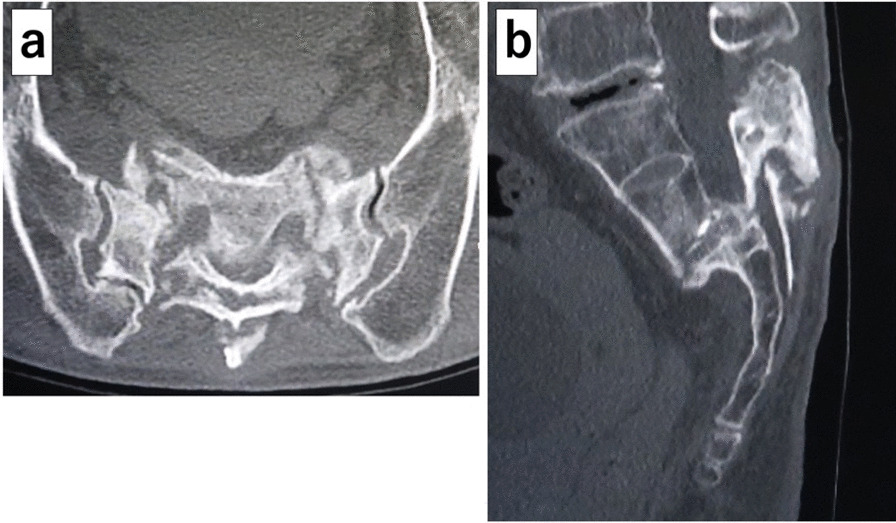
Preoperative computed tomography. **a** Sagittal reconstruction image showing the sacral fracture consisting of a transverse fracture with displacement of the third sacral vertebra. **b** Axial image showing vertical fractures of the bilateral sacral wings

**Fig. 3:**
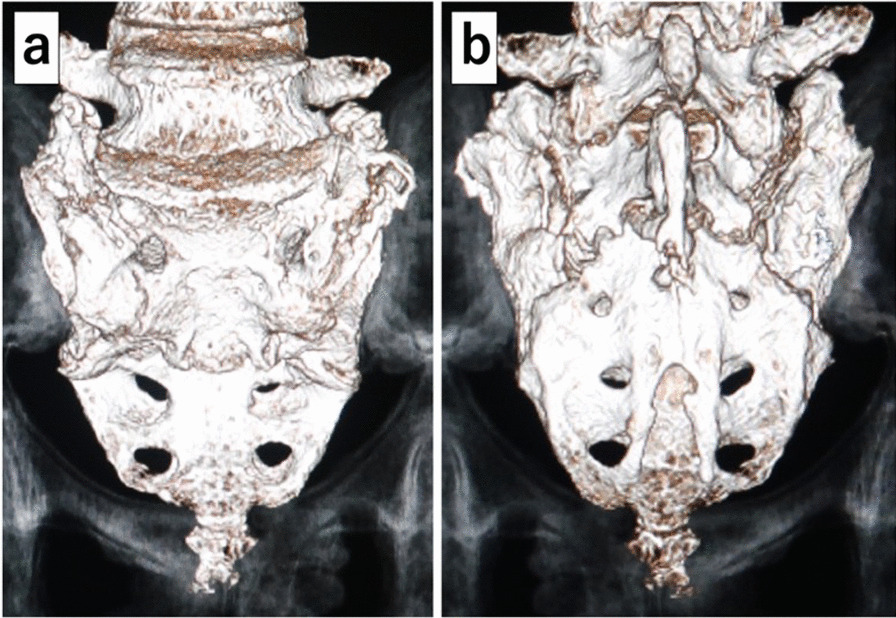
Preoperative three-dimensional computed tomography. **a** Frontal view, **b** Posterior view. Both views show an H-shaped sacral fracture (Rommens classification: type IVb)

**Fig. 4:**
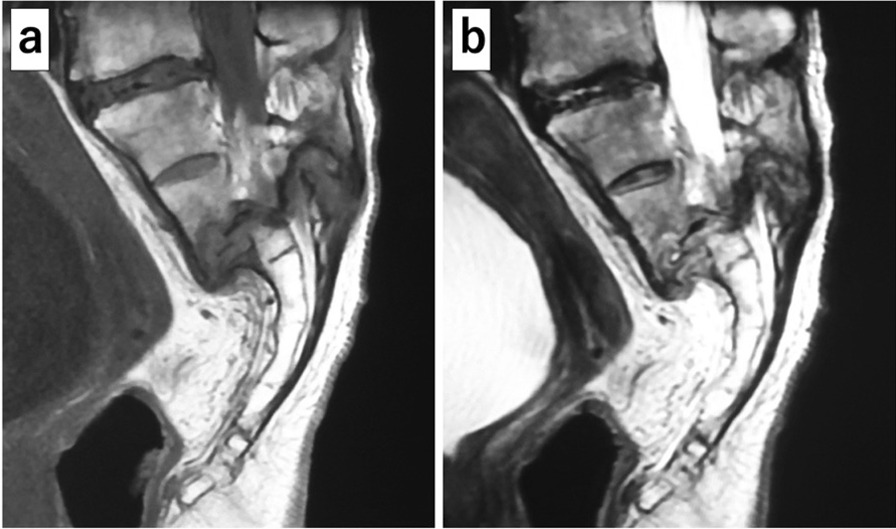
Preoperative MRI. **a** T1-weighted sagittal view, **b** T2-weighted sagittal view. Preoperative MRI showing severe stenosis of the spinal canal at the site of the transverse fracture

Her plain X-ray on admission to the orthopedic department showed a transverse fracture of the third sacral vertebra with displacement, which was not observed in the first X-ray of her lumbar spine on admission to the emergency department (Fig. 1b). An H-shaped sacral fracture (Rommens classification: type IVb) consisting of a transverse fracture with displacement of the third sacral vertebra and vertical fractures of the bilateral sacral wings was seen on computed tomography (CT) (Figs. 2a, b, 3). Magnetic resonance imaging (MRI) showed severe stenosis of the spinal canal at the site of the transverse fracture (Fig. 4). The patient was then diagnosed as having bladder and rectal dysfunction due to a displaced sacral fracture with instability, and she was treated surgically.

**Fig. 5:**
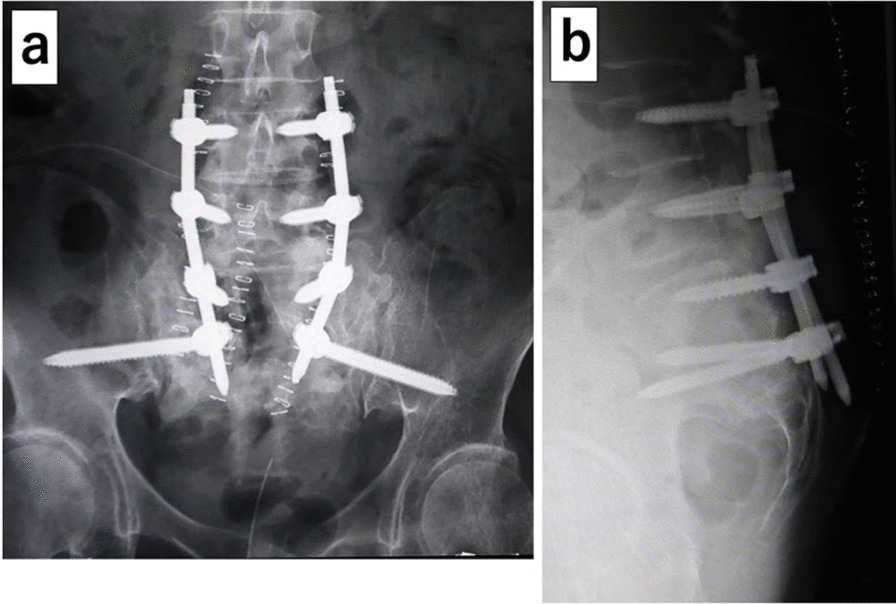
Postoperative radiographs. **a** Anteroposterior image; **b** lateral image. First to third sacral laminectomy and alar–iliac fixation using percutaneous pedicle screws and sacral alar–iliac screws have been performed

**Fig. 6:**
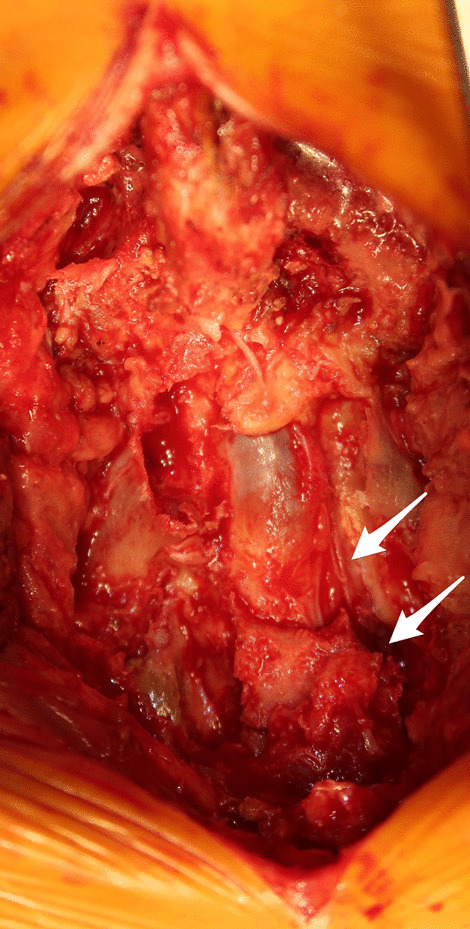
Intraoperative image. The bilateral distal sacral nerve roots have  been completely severed at the second to third sacral levels (white arrows)

**Fig. 7:**
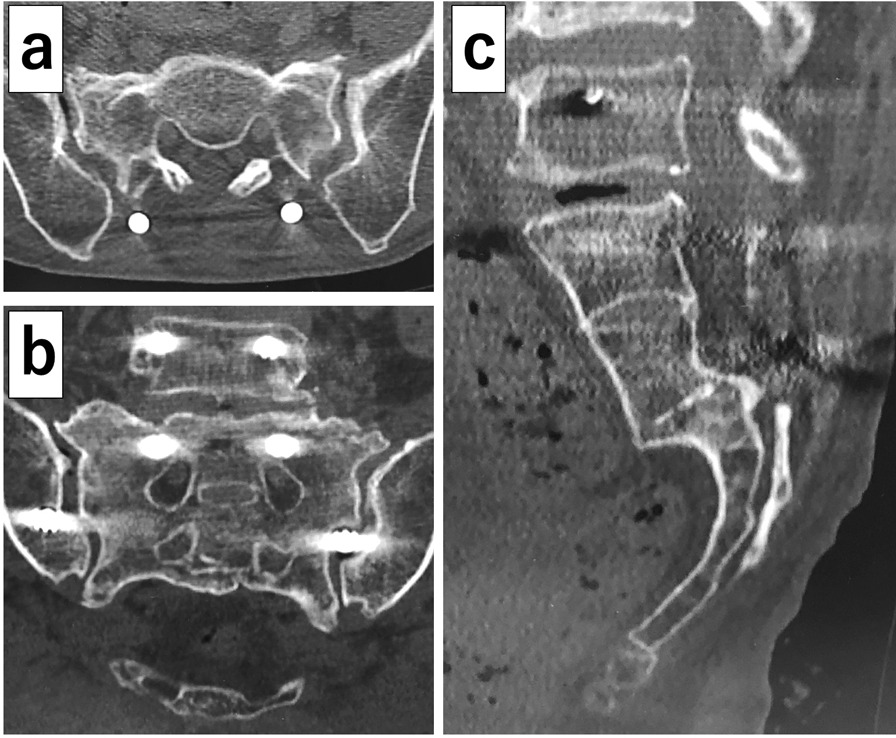
Postoperative computed tomography 3 months after surgery. **a** Axial image; **b** coronal reconstruction image; **c** sagittal reconstruction image. Bone fusion is observed in all images

First to third sacral laminectomy was performed, along with alar–iliac fixation using percutaneous pedicle screws and sacral alar–iliac screws (Fig. 5). Autogenous bone grafting using bone tissue from the sacrum removed by laminectomy was also performed to allow lumbosacral fixation. The bilateral distal sacral nerve roots (S3, S4, S5) were completely severed at the second to third sacral levels (Fig. 6), but the second sacral nerve roots on both sides were not compressed at the site from the bifurcation to the sacral foramen. After the operation, the bladder and rectal dysfunction remained, but the low back pain was alleviated. Two weeks after the operation, she could walk with a walker and was discharged. Three months after the operation, bone fusion of the fracture was observed, and no significant adverse events had occurred (Fig. 7).

## Discussion and conclusions

The symptoms and findings of sacral fracture include not only tenderness of the sacrum, but also low back pain, buttock pain, groin pain, and lower-extremity pain. Moreover, standing worsens the symptoms of this fracture [5]. This fracture may also cause neuropathy, such as bladder–rectal dysfunction or perianal hypoesthesia. The main diagnostic pitfalls in sacral fractures are that they are often overlooked or misdiagnosed as lumbar spine disease because the symptoms are similar to those of lumbar spine disease, and the diagnosis rate is low on plain X-ray [6, 7]. There is also a report of a case of an acute neurological deficit due to a sacral insufficiency fracture with coexistent lumbar canal stenosis [8]. Wagner *et al*. reported that the diagnostic rates with X-ray, CT, and MRI for sacral fractures were 0–10%, 60–75%, and 100%, respectively [8]. In the present case as well, although a plain X-ray of the lumbar spine was performed in the emergency department, the sacral fracture was not diagnosed. The sacral fracture was found on sagittal MRI of the lumbar spine, and additional MRI and CT of the pelvic area showed the details of the fracture in the present case. From the initial stage, the possibility of sacral, as well as lumbar spine, disease should have been considered in the differential diagnosis. Additional imaging studies of the sacrum should not be delayed in the absence of a lumbar spine lesion.

One of the symptoms of sacral fracture that should be specially noted is bladder–rectal dysfunction. Denis *et al*. reported that the probability of neuropathy due to sacral fracture was 5.9% in zone I, 28.4% in zone II, and 56.7% in zone III, and that a sacral fracture in zone III is the most prone to bladder–rectal dysfunction [3].

In the present case, the sacral fracture may have caused neuropathy from the beginning, since the patient had already had dysuria on the first visit to the emergency department. Then, the subsequent displacement of the fracture had made the neuropathy irreversible. The area of anesthesia also supported this explanation.

Rommens *et al*. classified FFP into four types, type I to type IV [4]. They also reported that sacral fractures accounted for about 50% of all FFP cases, and H-shaped sacral fractures classified as Rommens type IVb accounted for 15% of all cases [10]. There is a report that H-shaped sacral fractures accounted for 60% of all sacral fractures [11], indicating that they are not a rare type. Type IV is the most unstable type, especially type IVb, which includes transverse fractures of the sacrum, and it is classified as zone III in the Denis classification.

Conservative treatment is the first choice for types I and II, and surgery is also recommended for type II when the pain persists for several days despite conservative therapy. For types III and IV, surgery is recommended. Iliosacral screw fixation and lumbopelvic fixation are used mainly for H-shaped or U-shaped sacral fractures, and lumbopelvic fixation has been reported to be superior in the rate of return to home [12]. In other words, spinopelvic fixation is essential because of the discontinuity between the spine and pelvis [13, 14].

In addition, fracture reduction and laminectomy are required in cases with displacement and neuropathy [15, 16]. Percutaneous surgery can reduce blood loss and operative time in cases that do not require open reduction [17].

In the present case, lumbopelvic fixation and laminectomy were performed without reduction and fixation of the transverse fracture, because the bilateral sacral nerve roots were completely severed, and the neuropathy was considered to not be improving. The effectiveness of early surgical treatment for bladder function remains controversial [18]. However, it has been reported that surgical reduction and fixation of the displacement can prevent the pain due to residual displacement, prevent hematoma, preserve the remaining nerve, and provide early ambulation, even if severe nerve damage is observed [19]. In addition, angular deformity of the fracture, size of dislocation, and neurological deficits are factors associated with a poor prognosis [18]. Thus, the reduction and fixation of transverse fractures should be considered depending on the fracture type and neurological symptoms.

A case of distal sacral nerve roots severed by a displaced fragility fracture of the sacrum was described. Even sacral fractures without displacement require attention because they can cause serious injury in the event of a severed nerve root if not diagnosed early and given appropriate treatment.

## Data Availability

Not applicable.

## Abbreviations

FFPs: Fragility fractures of the pelvis
CT: Computed tomography
MRI: Magnetic resonance imaging
